# Projection to Latent Spaces Disentangles Pathological Effects on Brain Morphology in the Asymptomatic Phase of Alzheimer's Disease

**DOI:** 10.3389/fneur.2020.00648

**Published:** 2020-07-28

**Authors:** Adrià Casamitjana, Paula Petrone, José Luis Molinuevo, Juan Domingo Gispert, Verónica Vilaplana

**Affiliations:** ^1^Image and Video Processing Unit, Department of Signal Theory and Communications, UPCBarcelona Tech, Barcelona, Spain; ^2^Barcelonabeta Brain Research Center (BBRC), Pasqual Maragall Foundation, Barcelona, Spain; ^3^CIBER Fragilidad y Envejecimiento Saludable (CIBERFES), Madrid, Spain; ^4^Departament de Ciències Experimentals i de la Salut, Universitat Pompeu Fabra, Barcelona, Spain; ^5^CIBER de Bioengeniería, Biomateriales y Nanomedicina (CIBER-BBN), Madrid, Spain

**Keywords:** Alzheimer's disease, PLS, pre-clinical AD, latent model, CSF biomarkers, brain morphology

## Abstract

Alzheimer's disease (AD) continuum is defined as a cascade of several neuropathological processes that can be measured using biomarkers, such as cerebrospinal fluid (CSF) levels of Aβ, *p*-tau, and *t*-tau. In parallel, brain anatomy can be characterized through imaging techniques, such as magnetic resonance imaging (MRI). In this work we relate both sets of measurements and seek associations between biomarkers and the brain structure that can be indicative of AD progression. The goal is to uncover underlying multivariate effects of AD pathology on regional brain morphological information. For this purpose, we used the projection to latent structures (PLS) method. Using PLS, we found a low dimensional latent space that best describes the covariance between both sets of measurements on the same subjects. Possible confounder effects (age and sex) on brain morphology are included in the model and regressed out using an orthogonal PLS model. We looked for statistically significant correlations between brain morphology and CSF biomarkers that explain part of the volumetric variance at each region-of-interest (ROI). Furthermore, we used a clustering technique to discover a small set of CSF-related patterns describing the AD continuum. We applied this technique to the study of subjects in the whole AD continuum, from the pre-clinical asymptomatic stages all the way through to the symptomatic groups. Subsequent analyses involved splitting the course of the disease into diagnostic categories: cognitively unimpaired subjects (CU), mild cognitively impaired subjects (MCI), and subjects with dementia (AD-dementia), where all symptoms were due to AD.

## 1. Introduction

Alzheimer's disease (AD) is a neurodegenerative disease characterized by a progressive cognitive and memory decline and specific neuropathological processes, namely extracellular beta-amyloid plaque deposition and intracellular neurofibrillary tangles accumulation (1). Initial diagnostic criteria defined AD as a syndrome without neuropathological confirmation for a “probable AD diagnosis” and with confirmation for a “definite” one (2). However, during the last decade, a biological definition of AD describing specific neuropathological changes that could cause dementia has been introduced (3, 4), leading to the definition of the pre-clinical stage of AD (5). A recent paper (6) suggests a new research framework considering AD purely as a biological continuum, excluding clinical symptoms from its definition. According to this framework the term “Alzheimer's disease” is applied, regardless of the clinical manifestations, whenever there is evidence of pathologic deposits of both Aβ and tau in the brain, as manifested, for example, through AD cerebrospinal (CSF) core biomarkers: Abeta42, phosphorylated Tau (*p*-tau), and total Tau (*t*-tau) which reflect amyloid pathology, tau pathology, and neurofibrillary tangle neurodegeneration, respectively. When there is evidence of Aβ pathology but not tau, the term “Alzheimer's pathologic change” should be used. Together, individuals with either Alzheimer pathologic change or Alzheimer's disease belong to the so-called “Alzheimer's continuum”, and these individuals may be in different clinical stages, from the cognitively unimpaired stage to the stage of dementia. Similar to this criteria, the Food and Drug Administration (FDA) of the United States government proposed a 4-stage model (7) where stage 1 (no clinical impact) and stage 2 (subtle detectable abnormalities on sensitive neuropsychological measures) fall into the pre-AD category and stages 3 and 4 fall into the MCI and AD-dementia categories, respectively.

Quantitative MRI has broadly been used in AD (8) to show structural brain differences among groups of subjects in cross sectional studies [e.g., hippocampal neuronal loss between normal aging and subjects with dementia (9)] or to assess structural brain evolution in longitudinal studies [e.g., hippocampal brain atrophy rates in (10)]. As a measure of neurodegeneration, MRI is considered a valid marker of AD progression (11) and it is used in the clinical assessment of suspected AD patients (12) and is proposed for clinical screening applications (13, 14). Overall, brain morphometry is concerned with the study of physiological processes occurring in the brain that can be related to factors like aging and/or neurodegenerative diseases.

Projection to Latent structures (PLS), also known as Partial Least Squares, is a multivariate method that relates information from two different sets of measurements describing a single set of observations, by means of unobservable latent variables. These latent variables are derived from the input measurements that best explain the joint variation of both domains and are seen as associated morphological patterns. As a multivariate model, it simultaneously models the relationship between variables from the same set of measurements. It allows finding the relative contribution of each variable to the cross-domain effects and may uncover hidden effects from the respective univariate analysis. Out of several PLS methods, we used partial least squares correlations (PLSC) as a descriptive technique that maximizes the cross-covariance between latent spaces.

In this work, we studied the relationship between brain morphology and the two underlying processes of aging and AD pathology. The main aim is to discover morphological patterns associated to each process by disentangling their respective effects. CSF biomarkers (CSF Aβ, CSF *p*-tau, and CSF *t*-tau) are used as an *in-vivo* measure of AD pathology; age and sex are used as a proxy of brain aging, and volumetric MRI as a proxy of brain neurodegeneration. We used PLS analysis to relate the information obtained from CSF biomarkers and MRI, and to uncover common patterns that may be attributed to neurodegenerative processes due to AD. We utilized a preliminary model based on normal aging subjects that were perpendicular to the disease model.

### 1.1. PLS in Neuroimaging

PLS modeling, either in its regression (PLSR) or correlation (PLSC) variants, has been widely used in neuroimaging. It was first introduced in functional neuroimaging by McIntosh et al. in (15) and Krishnan et al. (16) provide a complete review of several subsequent applications in the field. In Ziegler et al. (17), PLSC was used to study the relationship between the cognitive profile and voxelwise MRI volumetric features in children and adolescents. In the same line, PLSC was used to model the interactions between genetic profile and MRI phenotypes in Lorenzi et al. (18). More recently, alterations in white matter due to Alzheimer's disease were studied in Konukoglu et al. (19) by examining the relationship of several diffusion maps with different AD stages. Finally, PLS has also been used as a feature extractor in a larger machine learning analysis pipeline (20).

Most studies integrating both CSF and MRI information, found in the literature, employ other modeling techniques. CSF biomarkers and MRI information have been used to discriminate between diagnostic AD stages (21) and to examine the ability of predicting the time to conversion to other stages in Vemuri et al. (22). Both works found similar results and conclude that CSF and MRI provide complimentary information in discriminative and predictive tasks, even though MRI outperforms CSF in both. The work in Fjell et al. (23) studied the relationship between CSF biomarkers and brain morphometry for assessing changes in brain structure between cognitively unimpaired (CU) subjects and subjects in symptomatic stages of AD (MCI, AD-dementia). They use the general linear model (GLM) to independently model each brain ROI with a single CSF biomarker and perform hypothesis testing to assess their relevance. They also studied the effect of CSF biomarkers in brain differences between groups (CU/MCI, CU/AD-dementia) using cortical thickness and volumetric information. Finally, the work in Casamitjana et al. (24) used PLSR to study the relationship between MRI and CSF along the disease continuum, finding common multivariate anatomical patterns along the AD that are predictive of CSF biomarkers.

## 2. Materials and Methods

### 2.1. Data

In our experiments we used the publicly available dataset from the Alzheimer's Disease Neuroimaging Initiative (ADNI^1^) with imaging data preprocessed using FreeSurfer. According to Sperling et al. (5), we split the AD continuum (*N* = 801) into three clinical stages: a total of *N*_CU_ = 321 cognitively unimpaired (CU) subjects, *N*_MCI_ = 332 subjects with mild cognitive impairment (MCI), and *N*_AD-dementia_ = 148 subjects diagnosed with dementia due to AD (AD-dementia). For symptomatic stages (MCI and AD-dementia), only amyloid positive subjects [CSF *Aβ* <192 pg/mL, (25)] were considered. A summary of the demographic information can be found in Table 1.

**Table 1:** Multivariate CSF effects on brain morphology along the AD continuum.

	**CU**	**MCI**	**AD**
Age	73.17 (± 6.20)	73.15 (±6.97)	74.83 (±7.84)
Education	16.84 (±2.45)	16.25 (±2.76)	15.85 (±2.65)
Sex (F/M)	170/151	142/190	64/84
# apoE4 (0/1/2)	227/85/9	116/162/54	39/74/35
MMSE	29.04 (±1.22)	27.64 (±1.96)	22.79 (±2.62)
FAQ	0.34 (±1.18)	3.21 (±4.12)	13.97 (±6.84)

*Statistical significant ROIs are grouped in 4 clusters characterized by their centroid. We refer to all centroids as the latent pathological patterns driving morphological changes and they are shown in the header of the table*.

All subjects have cortical and subcortical gray matter volumetric information (*K* = 88) available for each brain ROI (26, 27) as imaging measurements; age and sex as confounder variables and CSF biomarkers [*Aβ*, phosphorylated tau (*p*-tau), and total tau protein (*t*-tau)] as pathophysiological measurements. Typically, AD subjects show decreasing CSF Aβ values, increasing CSF *t*-tau/*p*-tau burden, and brain atrophy. Hence, we will refer to typical AD-related pathophysiological patterns as those underlying patterns characterized by a positive correlation between volumetric features and CSF Aβ and a negative correlation between volumetric features and CSF *t*-tau/*p*-tau.

### 2.2. Partial Least Squares Correlation Framework

Partial Least Squares Correlation (PLSC) is a statistical method that describes the relationship between two sets of measurements, X and Y, on the same observations. This relationship is modeled as the covariance between both input spaces (X, Y) and the goal is to examine their shared information. The underlying assumption of PLSC modeling is that most of the joint variability between X and Y lies in a lower dimensional space, i.e., it can be described by means of a few latent patterns.

Let us assume we have *N* subjects with two sets of different measurements: *K* descriptive variables $\text{X}\in R^{N\text{x}K}$ (e.g., brain structure using MRI) and *P* condition-related variables $\text{Y}\in R^{N\text{x}P}$ (e.g., CSF biomarkers). Without loss of generality, we assume both variables to be mean-centered. Formally, PLSC is applied to identify two new sets of variables $\text{T}\in R^{N\text{x}L}$, $\text{U}\in R^{N\text{x}L}$, called latent variables, which are linear combinations of the original measurements **X** and **Y**, respectively. These new variables lie in lower and unobserved *L*-dimensional spaces derived by simultaneous decomposition of input variables trying to maximize their cross-covariance (28). This idea is translated into finding the directions of maximum covariance between the original input spaces under the orthogonal constraint on the *L* projection vectors:

(1) $$\begin{array}{rc} \text{maximize} & \text{cov}\left(\text{X}{\text{w}}_{l},\text{Y}{\text{c}}_{l}\right)=\text{cov}\left({\text{t}}_{l},{\text{u}}_{l}\right)\\ \;\text{s.t} & {\text{w}}_{l}^{⊤}\Delta {\text{w}}_{l^{\prime }}=\delta \left(l-l^{\prime }\right),{\text{c}}_{l}^{⊤}\Delta {\text{c}}_{l^{\prime }}=\delta \left(l-l^{\prime }\right) \end{array}$$

where ${\text{w}}_{l}\in R^{K\text{x}1},{\text{c}}_{l}\in R^{P\text{x}1}$ are the projection weight matrices from input to latent spaces. It follows from the properties of singular value decomposition (SVD) that **w**_*l*_, **c**_*l*_ are the left and right singular vectors of the covariance matrix *R* = **X**^⊤^**Y** (29), respectively. Moreover, the covariance of the latent space at each dimension, i.e., cov(**t**_*l*_, **u**_*l*_), is equal to the corresponding singular value. The final *L*-dimensional latent space is built by concatenating the corresponding latent variables:

(2) $$\begin{array}{r} \;\text{T}=\left[{\text{t}}_{0},{\text{t}}_{1},\ldots ,{\text{t}}_{L-1}\right],\text{T}=\text{X}\text{W}\\ \;\text{U}=\left[{\text{u}}_{0},{\text{u}}_{1},\ldots ,{\text{u}}_{L-1}\right],\text{U}=\text{Y}\text{C}\\ \text{where}\text{W}=\left[{\text{w}}_{0},{\text{w}}_{1},\ldots ,{\text{w}}_{L-1}\right],\text{C}=\left[{\text{c}}_{0},{\text{c}}_{1},\ldots ,{\text{c}}_{L-1}\right] \end{array}$$

### 2.3. Model Definition

A common assumption in neuroimaging studies is that the object of study (e.g., brain morphology) is affected at the same time by the condition of interest (e.g., dementia) and confounding variables (e.g., age, genetics). Hence, we need to control for confounding variables in any neuroimaging analysis in order to find meaningful results. The standard solution is to regress-out the unwanted factors on the condition of interest. In a PLS framework, it can be done by estimating two separate models for confounders (*M*^*cAD*^) and the variable of interest (*M*^*AD*^) and imposing orthogonality between both models. Each model is characterized using two different Y-space variables (**Y**^*cAD*^, **Y**^*AD*^) and the same X-space. Similar to the work in Konukoglu et al. (19), we introduce the orthogonality constraint in the optimization process, forcing the associated latent subspace (**T**^*cAD*^, **T**^*AD*^) to be orthogonal.

First, the confounder model, *M*^*cAD*^, is estimated using MRI (**X**∈ℝ^*N*x*K*^) and age and sex **Y**^*cAD*^∈ℝ^*N*x2^) as predictor and response variables, respectively. The associated subspaces (**T**^*cAD*^, **U**^*cAD*^) are found by solving the regular expression in Equation (1) for an *L*^*cAD*^ = 1-dimensional subspace with weights ${w_{0}}^{cAD}$ and ${c_{0}}^{cAD}$.

Second, the model of interest, *M*^*AD*^, is estimated using deflated MRI ($\bar{X}\in {\text{R}}^{N\text{x}K}$) and CSF biomarkers (**Y**^*AD*^∈ℝ^*N*x3^) as predictor and response variables, respectively. To account for the variance explained by the confounder model, we define the new predictor variable

(3) $$\begin{array}{r} \bar{X}=X-\sum_{l=0}^{L^{cAD}-1}X\cdot \left(\frac{t_{1l}\cdot t_{1l}^{⊤}}{t_{1l}^{⊤}\cdot t_{1l}}\right) \end{array}$$

where we subtract the measurement variance explained by *M*^*cAD*^ and ensure orthogonality between subspaces. To study the effect at each ROI, we estimate *K* submodels ($M_{k}^{\text{AD}},\forall k=0,\ldots ,K-1$) each one using the deflated version of regional volumetric features for the *k*-th ROI (${\bar{X}}_{k}$) as predictor variables. For each submodel we set $L_{k}^{\text{AD}}=1$ yielding univariate structural model that describes regional effects of multivariate pathological effects. Each model is estimated by solving Equation (1) with weights ${{\text{w}}_{\text{k}}}^{cAD}$ and ${{\text{c}}_{\text{k}}}^{cAD}$.

### 2.4. Statistical Inference

The outcome measures of the estimated models are the *effect size* (ρ) and the *effect type* (ν_*X*_, ν_*Y*_). The effect size is a quantitative measure of the magnitude of a certain phenomenon, while the effect type is defined over multivariate phenomena as the vector of proportions indicating relative effect sizes of each parameter. Inherently, PLSC models have estimated two different latent subspaces, each one related to X and Y input spaces, respectively. Hence, a good definition for the effect size is the covariance between both estimated subspaces at each dimension, while the effect types are defined as the vectors of projection to the associated subspace

(4) $$\begin{array}{r} \rho_{l}=\frac{1}{N-1}{\text{t}}_{l}^{⊤}{\text{u}}_{l},\nu_{Xl}={\text{w}}_{l},\nu_{Yl}={\text{c}}_{l}\forall l=0,\ldots ,L-1 \end{array}$$

We report outcome measures for both PLS models (*M*^*cAD*^, *M*^*AD*^). For the confounder model, the effect size and effect type read as follows:

(5) $$\begin{array}{r} \rho^{\text{cAD}}=\frac{1}{N-1}t_{0}^{cAD^{⊤}}\cdot u_{0}^{cAD},\nu^{\text{cAD}}=\frac{1}{\rho }\cdot w^{cAD} \end{array}$$

In the model of interest, we define an effect size and effect type for each submodel:

(6) $$\begin{array}{l} \rho_{k}^{AD}=\frac{1}{N-1}t_{\text{k}}^{AD^{⊤}}\cdot u_{k}^{AD},\nu_{k}^{AD}=c_{k}^{AD},\text{where}\\ \;k=0,\ldots ,K-1 \end{array}$$

All effect types are normalized to unit norm ($||\nu_{k}^{AD}|{|}_{2}=1$) and each value corresponds to the relative contribution of each CSF biomarker on explaining the variance of ROI volumes.

#### 2.4.1. Non-parametric Permutation Testing

To assess the significance of the estimated effect size, non-parametric permutation testing is used (30). The null hypothesis states that there is no relationship between descriptive (*X*) and condition-related (*Y*) variables, hence, the effect size of the analysis is small/non-significant. Then, the null distribution of the effect size is estimated by randomly permuting subject indices in one measurement (i.e., *X*_π_) to break the initial relationship and generating a new sample of unrelated variables. This process is repeated N_perm_ times, and for each permutation (π(*i*), i=0, …, N_perm_−1) a new PLSC model is computed along with the associated effect size $\rho_{l}^{\pi \left(i\right)}$ at each dimension. For any $\rho \in \left(\rho^{cAD},\rho_{k}^{AD}\right)$, $t\in \left(t_{0}^{cAD},t_{k}^{AD}\right)$ and $u\in \left(u_{0}^{cAD},u_{k}^{AD}\right)$, the effect size at each permutation is calculated:

(7) $$\begin{array}{r} \rho_{l}^{\pi \left(i\right)}=\frac{1}{N-1}{\text{t}}_{\pi \left(i\right)}^{⊤}\cdot \text{u} \end{array}$$

where π(*i*) is the *i*-th permutation without replacement of subject indices. The null distribution is empirically built using $\rho^{\pi \left(i\right)},\forall i=0,\ldots ,{\text{N}}_{\text{perm}}$. Statistical significance level (*p*-value) of the observed effect size at each dimension (ρ) is determined by the ratio of permutations that result in a higher effect size.

(8) $$\begin{array}{r} p\text{-value}\left(\rho \right)=\frac{1}{N_{\text{perm}}}||\rho^{\pi \left(i\right)}>\rho |{|}_{0} \end{array}$$

where ||·||_0_ is the 0-norm operator, that counts the number of non-zero elements of a vector. In this work, we used a significance level of *p*-value < 0.05, corrected using a false discovery rate of 5% (FDR=0.05).

An important parameter for inference, using permutation testing, is the number of permutations (N_perm_) to estimate the null distribution. Larger N_perm_ provides better estimations but increases the computational cost. Hence, there is a trade-off between computational complexity and the precision (P) of the *p*-value(ρ) estimation. It is known that the Monte Carlo approximation of the *p*-value has a standard deviation of $\sqrt{\frac{p(1-p)}{{\text{N}}_{\text{perm}}}}$ involving the true real value of *p*. Since *p* is unknown, Ojala and Garriga (31) suggest using the upper bound $\sqrt{\frac{1}{{\text{N}}_{\text{perm}}}}$ and model *p*-value(ρ) as approximately Gaussian with standard deviation P, referred to as the precision of the estimate. Then, for a desired precision P, the minimum number of permutations is.

(9) $$\begin{array}{r} N_{\text{perm}}\geq \frac{1}{4\cdot P^{2}} \end{array}$$

### 2.5. Clustering

Unsupervised clustering techniques allow uncovering common characteristics between sets of data without the need of a specific labeling process (32). In this work, the features of interest are the CSF biomarker characteristic patterns on brain morphology $mathbf\nu_{k}^{AD}$. Using the k-means algorithm (32) and the *M*^AD^ model, we aimed to group together different brain ROIs with similar effect types ($\nu_{k}^{AD}$). Hence, a total of *C* clusters, driven by their centroid (latent pattern) were found, uncovering underlying patterns of CSF biomarkers related to brain structure.

### 2.6. Ethics Committee Approval

All procedures performed in the ADNI studies involving human participants were in accordance with the ethical standards of the institutional research committees and with the 1964 Helsinki declaration and its later amendments. Written informed consent was obtained from all participants or their authorized representatives.

The study procedures were approved by the institutional review boards of all participating centers (https://adni.loni.usc.edu/wp-content/uploads/how__to__apply/ADNI__Acknowledgement__List.pdf): Oregon Health and Science University; University of Southern California; University of California—San Diego; University of Michigan; Mayo Clinic, Rochester; Baylor College of Medicine; Columbia University Medical Center; Washington University, St. Louis; University of Alabama at Birmingham; Mount Sinai School of Medicine; Rush University Medical Center; Wien Center; Johns Hopkins University; New York University; Duke University Medical Center; University of Pennsylvania; University of Kentucky; University of Pittsburgh; University of Rochester Medical Center; University of California, Irvine; University of Texas Southwestern Medical School; Emory University; University of Kansas, Medical Center; University of California, Los Angeles; Mayo Clinic, Jacksonville; Indiana University; Yale University School of Medicine; McGill University, Montreal-Jewish General Hospital; Sunnybrook Health Sciences, Ontario; U.B.C. Clinic for AD & Related Disorders; Cognitive Neurology—St. Joseph's, Ontario; Cleveland Clinic Lou Ruvo Center for Brain Health; Northwestern University; Premiere Research Inst (Palm Beach Neurology); Georgetown University Medical Center; Brigham and Women's Hospital; Stanford University; Banner Sun Health Research Institute; Boston University; Howard University; Case Western Reserve University; University of California, Davis—Sacramento; Neurological Care of CNY; Parkwood Hospital; University of Wisconsin; University of California, Irvine—BIC; Banner Alzheimer's Institute; Dent Neurologic Institute; Ohio State University; Albany Medical College; Hartford Hospital, Olin Neuropsychiatry Research Center; Dartmouth-Hitchcock Medical Center; Wake Forest University Health Sciences; Rhode Island Hospital; Butler Hospital; UC San Francisco; Medical University South Carolina; St. Joseph's Health Care Nathan Kline Institute; University of Iowa College of Medicine; Cornell University and University of South Florida: USF Health Byrd Alzheimer's Institute.

## 3. Results

In this article we analyzed the relationship between brain morphology and markers of normal aging and AD, along the AD continuum. Using the PLSC framework presented in section 2.2, we disentangled morphological patterns describing AD pathology measured using CSF biomarkers. Concretely we aimed to:

- Find multivariate patterns relating confounder variables and brain volumetry, and used them to regress-out the effect of confounders on brain morphology
- Describe the effect of CSF biomarkers in different volumetric brain ROIs.
- Look for specific patterns across the AD continuum, in different cognitive profiles (CU, MCI, AD-dementia).

### 3.1. Study of Age Association With Brain Morphometric Features in Normal Aging

**Figure 1:**
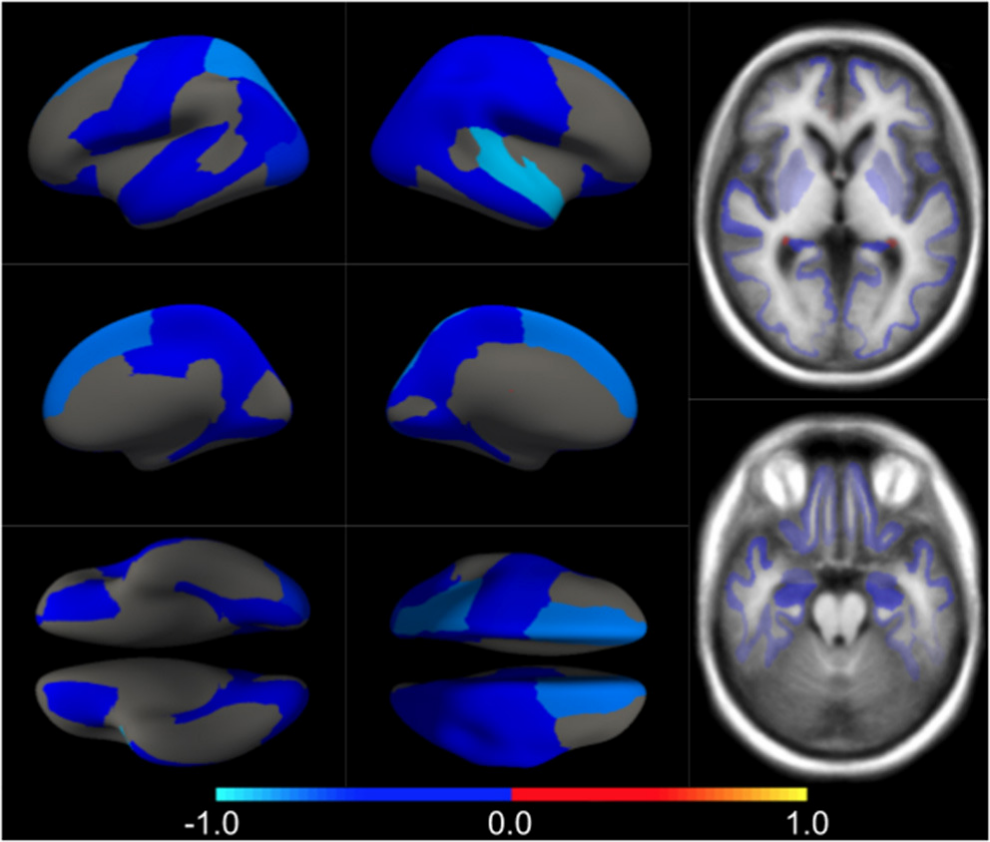
Different views of the multivariate brain morphometric effect of age (type *v*^*cAD*^). For visualization purposes, we have scaled the effect-type in the range [−1, 1] and masked all regions below a certain threshold (20% of the maximum value). The color-code represents negative (blue) and positive (red) relative contributions of each brain ROI to explaining the variability of confounders (age and sex).

We first estimated the aging model (M^cAD^) to regress-out the effect size of confounders (age and sex) on brain morphology in a posterior analysis. In Figure 1 we show the confounders' effect type, representing the multivariate pattern of volumetric variation related to age and sex. M^cAD^ model, as a proxy to the normal aging process, involves reduced cortical thickness in the whole brain with increasing age, with exceptions on the choroid plexus and the anterior cingulate. Regions that show a higher decrease in volume with age are found in the temporal lobe, especially the hippocampus. The sex effect size is rather low in the whole brain.

### 3.2. Study of CSF Biomarkers Association With Brain Morphometric Features Along AD Continuum

**Figure 2:**
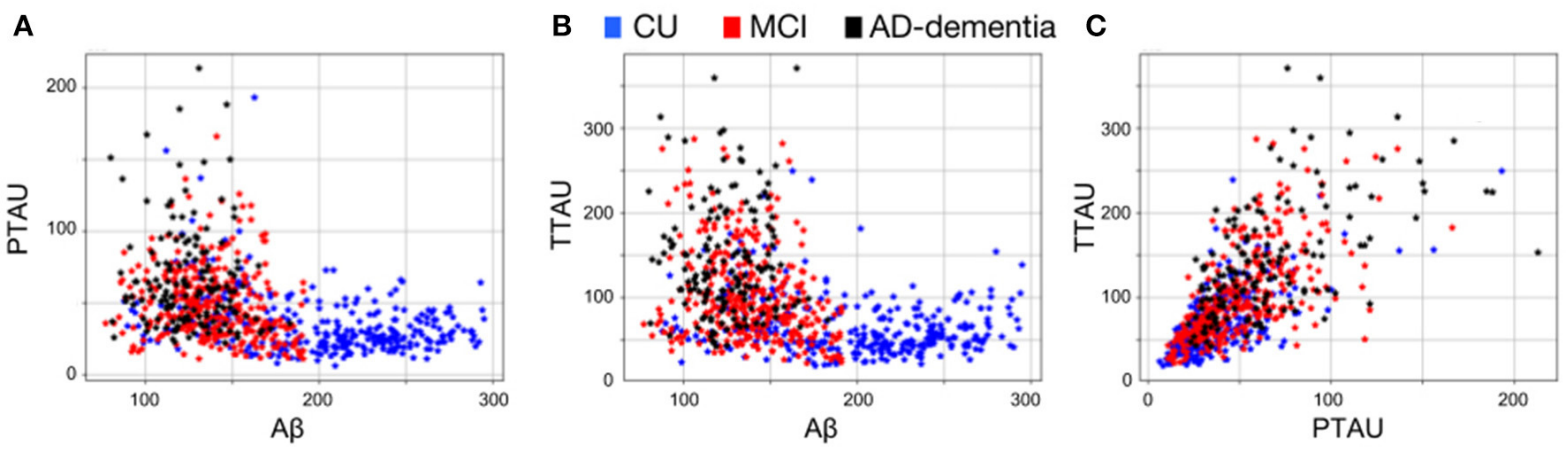
Pairwise CSF biomarker relationship along the AD continuum: **(A)** CSF Aβ vs. *p*-tau, **(B)** CSF Aβ vs. *t*-tau and **(C)** CSF *p*-tau vs. *t*-tau. Each point represents a subject and different colors refer to different clinical categories: CU (blue), MCI (red), and AD-dementia (black).

AD pathological effects are thought to be spread non-uniformly across the brain (33) and may only be described by a small set of patterns along the disease. Moreover, the non-linear aspect of a pairwise relationship between CSF *Aβ* and phosphorylated and total tau protein (Figure 2) may indicate that different patterns of brain volumetric variability coexist along the AD continuum. In this set of experiments, we aimed to capture these specific patterns using the AD model (M^AD^) and a clustering algorithm.

**Figure 3:**
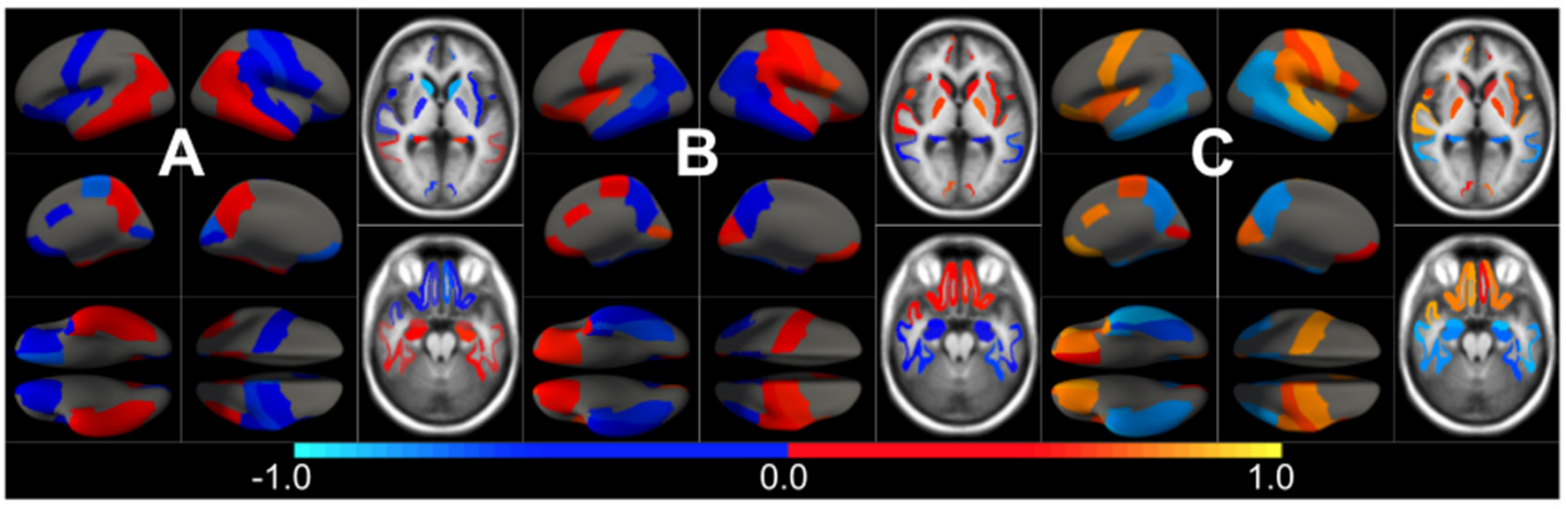
Different views of the effect of AD ($\nu_{k}^{AD}$) in subjects along the AD continuum. The color-code represents negative (blue) and positive (red) relative contributions of each CSF biomarker explaining the volumetric variability on brain each ROIs. Each figure represents a different CSF biomarkers: **(A)** CSF Aβ, **(B)** CSF *p*-tau, **(C)** CSF *t*-tau. Only brain ROIs with statistically significant effect size (*p* < 0.05, corrected for multiple comparisons) are shown.

In Figure 3, we show the condition-related effect type ($\nu_{k}^{AD}$) in statistically significant regions (*p* < 0.05, corrected for multiple comparisons). Each effect type value is split into different subfigures. A complete list of regional AD effect sizes and their related *p*-values can be found in Supplementary Table 1.1.

We then fit a clustering algorithm to find a small set of representative CSF-patterns that group all effect types ($\nu_{k}^{AD}$) across the brain and along disease stages. It effectively results in four different clusters with the corresponding centroid representing patterns of AD pathology on brain morphology. In Table 2, we provide a list of the relevant ROIs associated to each cluster. Hence, we can define four different underlying processes governing brain morphology. CSF *t*-tau appears to explain most of the variability in many brain ROIs (clusters 0 and 1). A typical AD-related pathophysiological pattern is found in cluster 1, where amyloid plaque deposition appears to favor the presence of tau protein in several temporal regions (hippocampus, inferior temporal, superior temporal, middle temporal, amygdala, fusiform and entorhinal cortex) and other typical AD regions (precuneus). In contrast, in cluster 0, several regions, such as pallidum, precental, lateral orbitofrontal, or precentral appear to develop compensation effects once discounting the aging effect with tau accumulation. On the other hand, CSF Aβ levels appear to highly affect regions of the caudate and other medial regions, such as the paracentral and the post-central, showing a compensatory mechanism with amyloid deposition once corrected by the aging process (clusters 3). Finally, the choroid plexus appears to be highly correlated with variation in CSF Aβ and *t*-tau values.

**Table 2:** Multivariate CSF effects on brain morphology for different diagnostic categories.

	**Centroid 0**	**Centroid 1**	**Centroid 2**	**Centroid 3**
	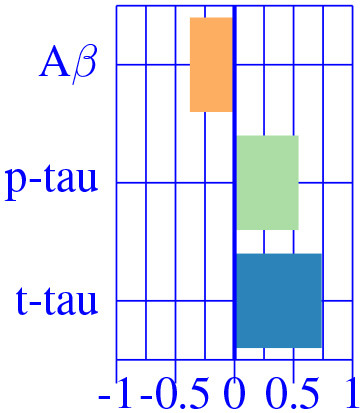	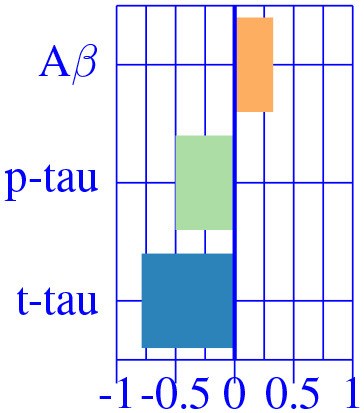	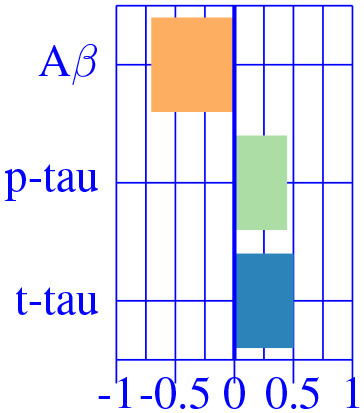	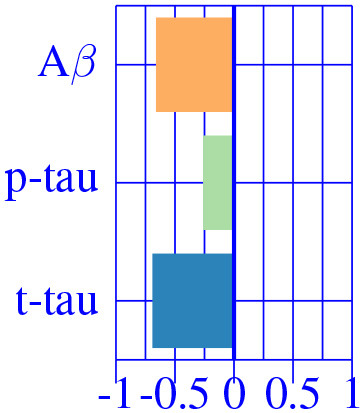
ADc	Pallidum R	Precuneus R	Cuneus L	Choroid Plexus R
	Pallidum L	Precuneus L	Pericalcarine R	Choroid Plexus L
	Precentral L	Amygdala R	Caudate R	
	Precentral R	Amygdala L	Caudate L	
	Lateral Orbitofrontal L	Bankssts R	Paracentral R	
	Lateral Orbitofrontal R	Bankssts L	Post-central R	
	Frontal Pole L	Entorhinal R	Medial Orbitofront. L	
	Frontal Pole R	Entorhinal L		
	Superior Temporal R	Fusiform R		
	Supramarginal R	Fusiform L		
	Caudal Ant. Cingulate R	Hippocampus R		
	Medial Orbitofrontal R	Hippocampus L		
	ParsOrbitalis L	Inferior Temporal R		
	Pericalcarine L	Inferior Temporal L		
	Temporal Pole L	Middle Temporal R		
	Transverse Temporal L	Middle Temporal L		
	Insula L	Inferior Parietal R		
	ParsOpercularis R	Inferior Parietal L		

*Statistical significant ROIs are grouped in 4 clusters characterized by their centroid. We refer to all centroids as the latent pathological patterns driving morphological changes and are shown in the header of the table*.

### 3.3. Study of CSF Biomarkers Association With Brain Morphometric Features in Different AD Stages

As suggested by Tosun et al. (33), in this second stage of analysis we considered that AD effects might be different along the disease continuum. Hence, we fit the corrected M^AD^ model independently to each diagnostic groups (CU, MCI and AD-dementia) and provided a *post-hoc* comparison.

**Figure 4:**
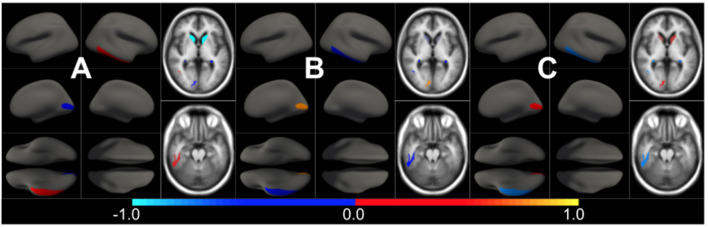
Different views of the effect of AD ($\nu_{k}^{AD}$) in subjects in the CU stage. The color-code represents negative (blue) and positive (red) relative contributions of each CSF biomarker explaining the volumetric variability on brain each ROIs. Each figure represents a different CSF biomarkers: **(A)** CSF Aβ, **(B)** CSF *p*-tau, **(C)** CSF *t*-tau. Only brain ROIs with statistically significant effect size (*p* < 0.05, corrected for multiple comparisons) are shown.

**Figure 5:**
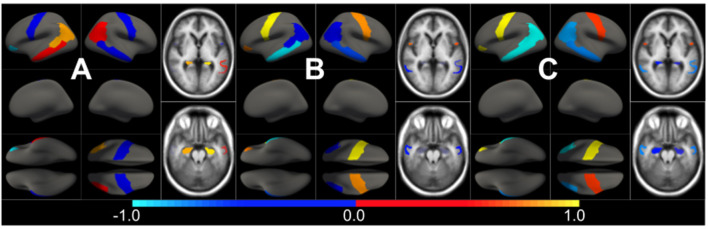
Different views of the effect of AD ($\nu_{k}^{AD}$) in subjects in the MCI stage. The color-code represents negative (blue) and positive (red) relative contributions of each CSF biomarker explaining the volumetric variability on brain each ROIs. Each figure represents a different CSF biomarkers: **(A)** CSF Aβ, **(B)** CSF *p*-tau, **(C)** CSF *t*-tau. Only brain ROIs with statistically significant effect size (*p* < 0.05, corrected for multiple comparisons) are shown.

The effect type for CU, MCI is shown in Figures 4, 5, respectively, in statistically significant regions while (*p* < 0.05, corrected for multiple comparisons) no significant regions were found for the AD-dementia stage once corrected for multiple comparisons. The *p*-values for each brain ROI are listed in Supplementary Table 2.1. Several subcortical and temporal pole regions appear to be highly correlated to AD pathophysiological markers at CU and especially MCI stages.

The resulting three-dimensional effect types can be effectively clustered into four different characteristic CSF patterns. In Table 3, we provide a list of relevant ROIs associated to the four centroids describing each cluster. Among the four clusters, we found that clusters 0 and 1, group regions whose variance is related to CSF *t*-tau/*p*-tau proteins variation, while clusters 2 and 3 appeared to be described by CSF Aβ load.

**Table 3:** Diagnostic categories.

	**Centroid 0**	**Centroid 1**	**Centroid 2**	**Centroid 3**
	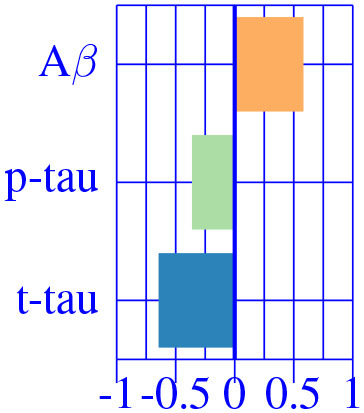	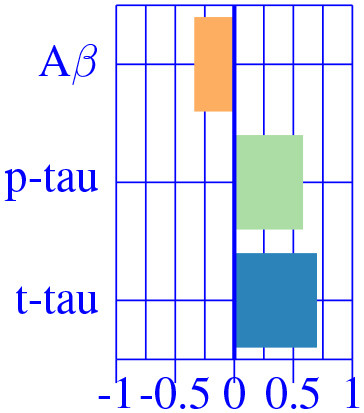	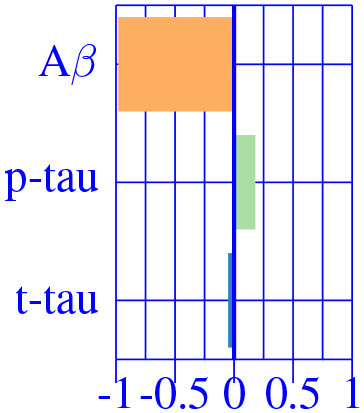	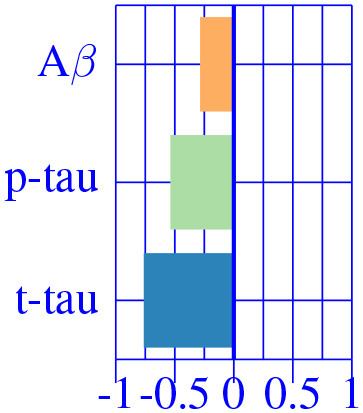
CU	LeftAccumbensArea L	RightPericalcarine	LeftCaudate	LeftChoroidPlexus
	RightInferiorTemporal		RightCaudate	RightChoroidPlexus
MCI	Banksts L	Precentral R		MidTemporal R
	MidTemporal L	ParsOrbitalis L		
	InferiorParietal R	Precentral L		
	InferiorParietal L			
	Hippocampus L			
	Hippocampus R			
AD				

A typical AD-related pathophysiological pattern is found in cluster 0 describing the variability of typical AD regions (inferior parietal and hippocampus) at intermediate stages (MCI). In contrast, cluster 1 shows a compensatory effect in medial regions (e.g., pre-central) at MCI stage. The caudate appears to be almost perfectly correlated with CSF Aβ values at early stages (CU), increasing its volume for decreasing CSF Aβ values. Finally, the pattern related to choroid plexus, shown in the previous sections, is found relevant only on cognitively unimpaired subjects. None of the regions below the significance threshold survive the multiple comparisons correction at late stages of the disease (AD-dementia).

## 4. Discussion

In this work we report the effect of normal aging and AD pathological processes on brain morphology. Age and sex were used as covariates in the normal aging model based on a cognitively unimpaired, amyloid-beta negative population that shows the standard pattern of global volume reduction (34) except for the choroid plexus. Its associated latent space was used as a confounding factor model to correct the disease model. AD pathology was measured using CSF biomarkers and shows high effect sizes on brain morphology along the disease spectrum. Brain structure can be effectively described by a small set of underlying patterns correlated with CSF biomarkers. Once corrected by the confounder model, they can be split into atrophy and volume compensation mechanisms.

A typical AD-related pathophysiological process is defined by cerebral atrophy in subjects with higher CSF *t*-tau and *p*-tau and lower CSF Aβ values. This pattern is most prominently found in the temporal lobe (hippocampus, superior, middle and inferior temporal, amygdala, fusiform and entorhinal cortex) as well as other regions, such as the pre-cuneus and the inferior parietal, all of them being established AD-vulnerable structures in the literature and used as diagnostic markers. Tau accumulation drives most of the volumetric variability on those regions. On the other hand, increased mean ROI volume, with increasing tau or decreasing amyloid-beta levels, is also present across the brain. This compensation mechanism is found in many regions, especially in the frontal lobe and in central regions, such as the precentral, the pallidum, or the caudate nucleus. Even though it does not belong to the central nervous system (CNS), a well-deserved remark should be made for the choroid plexus, understood to be the region that mediates CSF production and that seems independent of CSF *p*-tau levels, indicating its non-specific character for AD.

A subsequent analysis using clinical diagnosis to find group-specific effects shows different patterns along AD stages. Statistically significant regions at each stage drastically diminish compared to effects along the whole AD continuum. At pre-symptomatic stages of AD (i.e., cognitively unimpaired (CU) subjects), there exists a strong negative correlation between the caudate nucleus volume and CSF Aβ levels and between the choroid plexus and CSF *t*-tau values. This pattern appears to be specific for the asymptomatic stage while other regions present only mild effects. The number of regions that present relevant correlation between CSF biomarkers and brain morphology increases at MCI stage. A typical AD-related pathophysiological pattern is found in temporal lobe regions as well in other AD-vulnerable regions, such as the precentral and the inferior parietal, confirming previous findings in the literature [e.g., (35)]. This process is found in similar but fewer regions compared to the analysis on the whole AD continuum, indicating the spatial and temporal heterogeneity of the AD-signature. The joint variability between disease biomarkers and brain morphology is maximized at MCI stage, while at dementia stages this association is completely lost. This result shows that MCI stage is particularly interesting to study brain tissue deterioration due to Alzheimer's disease, while no clear pattern is found at early and late stages of the AD continuum.

There are some limitations to this work. First, we report preliminary results on the ADNI cohort and validation on an independent cohort would be required to assess the generalization of the results to other datasets as well as to the general population. Second, the oversimplification on the number of brain processes occurring in healthy adults' brain, was grossly split into AD and non-AD processes. Age and sex are used as the main confounders for non-AD effects while we acknowledge that many other factors (e.g., environmental, genetic) might be added to the model (36). CSF biomarkers are used as a proxy for AD effects considering that amyloidosis, tauopathy, and neurodegeneration drive AD pathology. However, many other comorbidities might be found in AD subjects, especially at later stages (37). Nonetheless, the work provides a methodology for the analysis of joint variation of imaging and non-imaging features, and results, consistent with the literature were found. New insights on brain morphology along the AD continuum are also reported.

## 5. Conclusion

In this work studied the relationship between CSF biomarkers and brain morphology that can be interpreted as the effect of abnormal amyloid and tau levels in the brain. Unlike standard hypothesis testing, PLS methods allow us to study normal aging and AD effects on brain morphology using continuous markers for both processes, instead of using a single categorical variable. To describe AD stages, we used age and sex as confounders for the normal aging model and CSF biomarkers to describe the AD continuum. In order to specifically analyze the effect regarding Alzheimer's disease, we superimposed a condition by which brain morphometry changes due to AD are properly orthogonalized to those due to normal brain aging. Concretely, we used Partial Least Squares Correlation (PLSC) to jointly describe patterns of change in CSF and MRI by projecting both spaces into correlated latent spaces. We found that CSF values are relevant to describe brain morphology along the AD continuum, both positively and negatively related to ROI volumetric features. This relationship appears to be maximal at MCI stage, while insignificant at late stages of dementia. CSF *p*-tau and *t*-tau appear to drive most of the variability associated to pathological processes being lined up with CSF Aβ. Overall, we proposed a statistically robust framework that can unravel hidden correlations between different measures of disease progression, to characterize neurodegenerative processes that govern brain morphology along the AD continuum.

## Data Availability Statement

The datasets used for this study can be found in the ADNI repository (adni.loni.usc.edu).

## Ethics Statement

This manuscript uses ADNI data. Ethical procedures can be found at the ADNI repository (adni.loni.usc.edu).

## Author Contributions

AC designed the methodology, performed the data analysis, and prepared the manuscript. VV helped in designing the methods and in writing the manuscript in a rigorous and clean way. PP, JM, and JG aided in discussing the results and revised and approved the manuscript. All authors contributed to the article and approved the submitted version.

### Conflict of Interest

The authors declare that the research was conducted in the absence of any commercial or financial relationships that could be construed as a potential conflict of interest.

## Footnotes

^1^http://adni.loni.usc.edu
