# Supplementary material for: Projection to Latent Spaces Disentangles Pathological Effects on Brain Morphology in the Asymptomatic Phase of Alzheimer's Disease
Source: Front Neurol. 2020 Jul 28;11:648. doi: 10.3389/fneur.2020.00648 (PMC7399334; doi:10.3389/fneur.2020.00648)
Supplement: Supplementary file 1 [file Data_Sheet_1.PDF]

Supplementary Material

1 LIST OF RELEVANT REGIONS ALONG THE AD CONTINUUM

Relevant regions list	
ROI name	AD continuum
Pallidum R	0.0005
Pallidum L	0.0014
Paracentral R	0.0112
Paracentral L	
Parahippocampal R	-
Parahippocampal L	-
ParsOperuclaris R	0.001
ParsOperuclaris L	-
ParsOrbitalis R	-
ParsOrbitalis L	0.0
ParsTriangularis R	-
ParsTriangularis L	-
Pericalcarine R	0.0031
Pericalcarine L	0.0
Postcentral R	0.0012
Postcentral L	-
PosteriorCingulate R	-
PosteriorCingulate L	-
Precentral R	0.0
Precentral L	0.0
Precuneus R	0.0001
Precuneus L	0.0034
Putamen R	-
Putamen L	-
RostralAnteriorCingulate R	-
RostralAnteriorCingulate L	-
RostralMiddleFrontal R	-
RostralMiddleFrontal L	-
SuperiorFrontal R	-
SuperiorFrontal L	-
SuperiorParietal R	-
SuperiorParietal L	-
SuperiorTemporal R	0.0064
SuperiorTemporal L	-
Supramarginal R	0.023
Supramarginal L	-
TemporalPole R	-
TemporalPole L	0.0042

Relevant regions list	
ROI name	AD continuum
Thalamus R	-
Thalamus L	-
TransverseTemporal R	-
TransverseTemporal L	0.0008
Insula R	-
Insula L	0.0002
AccumbensArea R	-
AccumbensArea L	-
Amygdala R	0.0
Amygdala L	0.0
Bankssts R	0.0045
Bankssts L	0.0
CaudalAnteriorcingulate R	0.0211
CaudalAnteriorcingulate L	-
CaudalMiddleFrontal R	-
CaudalMiddleFrontal L	-
Caudate R	-
Caudate L	0.0029
ChoroidPlexus R	0.0028
ChoroidPlexus L	0.0013
Cuneus R	0.0028
Cuneus L	0.0027
Entorhinal R	0.0
Entorhinal L	0.0
FrontalPole R	0.0006
FrontalPole L	0.0233
Fusiform R	0.0084
Fusiform L	0.0093
Hippocampus R	0.0
Hippocampus L	0.0
InferiorParietal R	0.0
InferiorParietal L	0.0
InferiorTemporal R	0.0006
InferiorTemporal L	0.0
IsthmusCingulate R	-
IsthmusCingulate L	-
LateralOccipital R	-
LateralOccipital L	-
LateralOrbitofrontal R	0.0
LateralOrbitofrontal L	0.0
Lingual R	-
Lingual L	-
MedialOrbitofrontal R	0.007

Relevant regions list	
ROI name	AD continuum
MedialOrbitofrontal L	0.0161
MiddleTemporal R	0.0
MiddleTemporal L	0.0

Table 1.1: List of brain ROIs ($p<0.05$, uncorrected) and their associated p-value in the AD continuum analysis of the CSF effect on mean ROI volume

2 LIST OF RELEVANT REGIONS IN DIFFERENT AD CONTINUUM STAGES.

Relevant regions list			
ROI name	CU	MCI	AD
Pallidum R	-	-	-
Pallidum L	-	-	-
Paracentral R	-	-	-
Paracentral L	-	-	-
Parahippocampal R	-	-	-
Parahippocampal L	-	-	-
ParsOperuclaris R	-	-	-
ParsOperuclaris L	-	-	-
ParsOrbitalis R	-	-	-
ParsOrbitalis L	-	0.0021	-
ParsTriangularis R	-	-	-
ParsTriangularis L	-	-	-
Pericalcarine R	0.0006	-	-
Pericalcarine L	-	-	-
Postcentral R	-	0.0	-
Postcentral L	-	-	-
PosteriorCingulate R	-	-	-
PosteriorCingulate L	-	-	-
Precentral R	-	-	-
Precentral L	-	0.0037	-
Precuneus R	-	-	-
Precuneus L	-	-	-
Putamen R	-	-	-
Putamen L	-	-	-
RostralAnteriorCingulate R	-	-	-
RostralAnteriorCingulate L	-	-	-
RostralMiddleFrontal R	-	-	-
RostralMiddleFrontal L	-	-	-
SuperiorFrontal R	-	-	-
SuperiorFrontal L	-	-	-
SuperiorParietal R	-	-	-

Relevant regions list			
ROI name	CU	MCI	AD
SuperiorParietal L	-	-	-
SuperiorTemporal R	-	-	-
SuperiorTemporal L	-	-	-
Supramarginal R	-	-	-
Supramarginal L	-	-	-
TemporalPole R	-	-	-
TemporalPole L	-	-	-
Thalamus R	-	-	-
Thalamus L	-	-	-
TransverseTemporal R	-	-	-
TransverseTemporal L	-	-	-
Insula R	-	-	-
Insula L	-	-	-
AccumbensArea R	-	-	-
AccumbensArea L	0.0018	-	-
Amygdala R	-	-	-
Amygdala L	-	-	-
Bankssts R	-	-	-
Bankssts L	-	0.0006	-
CaudalAnteriorcingulate R	-	-	-
CaudalAnteriorcingulate L	-	-	-
CaudalMiddleFrontal R	-	-	-
CaudalMiddleFrontal L	-	-	-
Caudate R	0.0014	-	-
Caudate L	0.0012	-	-
ChoroidPlexus R	0.0	-	-
ChoroidPlexus L	0.002	-	-
Cuneus R	-	-	-
Cuneus L	-	-	-
Entorhinal R	-	-	-
Entorhinal L	-	-	-
FrontalPole R	-	-	-
FrontalPole L	-	-	-
Fusiform R	-	-	-
Fusiform L	-	-	-
Hippocampus R	-	0.0023	-
Hippocampus L	-	0.0016	-
InferiorParietal R	-	0.0003	-
InferiorParietal L	-	0.0	-
InferiorTemporal R	0.0035	-	-
InferiorTemporal L	-	-	-
IsthmusCingulate R	-	-	-
IsthmusCingulate L	-	-	-

Relevant regions list			
ROI name	CU	MCI	AD
LateralOccipital R	-	-	-
LateralOccipital L	-	-	-
LateralOrbitofrontal R	-	-	-
LateralOrbitofrontal L	-	-	-
Lingual R	-	-	-
Lingual L	-	-	-
MedialOrbitofrontal R	-	-	-
MedialOrbitofrontal L	-	-	-
MiddleTemporal R	-	0.0004	-
MiddleTemporal L	-	0.0	-

Table 2.1: List of brain ROIs ($p<0.05$, uncorrected) and their associated p-value of the CSF effect on mean ROI volume. The analysis is performed independently on different categories along the AD continuum
